# Self-Assembly 4-Butylresorcinol Deep Eutectic Solvent Nanoparticles for Efficient Transdermal Delivery and Whitening

**DOI:** 10.3390/ph18091383

**Published:** 2025-09-16

**Authors:** Hongtao Han, Dan Hu, Yaoming Deng, Jiayi Song, Yuyang Sheng, Jingxin Liu, Chengyu Wu, Bin Zeng

**Affiliations:** College of Pharmacy, Shenzhen Technology University, Shenzhen 518118, China; 2410265081@mails.szu.edu.cn (H.H.); hudan@sztu.edu.cn (D.H.); 2410265073@stumail.sztu.edu.cn (Y.D.); 2201459@stu.neu.edu.cn (J.S.); yuyangsheng0519@163.com (Y.S.); liujingxin@sztu.edu.cn (J.L.)

**Keywords:** deep eutectic solvent, nanoparticles, self-assembly, enhanced transdermal delivery, whitening

## Abstract

**Background:** Skin whitening agents often face challenges such as poor stability and low permeability. To overcome these issues, a novel 4-butylresorcinol (4-BR)/D-a- Tocopherol Polyethylene Glycol Succinate (TPGS) deep eutectic solvent (DES) system was developed, which can self-assemble into carrier-free nanoparticles (NPs). **Methods**: The 4-BR/TPGS DES was synthesized and characterized by theoretical calculations, DSC, FTIR, 1H-NMR, and 2D NMR to confirm its successful formation. **Results**: The self-assembled 4-BR/TPGS DES NPs showed a 3.46-fold increase in skin permeability, a 1.53-fold improvement in 4-BR stability, a 1.55-fold increase in melanin inhibition in B16 cells, and a 2.16-fold higher melanin suppression in zebrafish compared with traditional 4-BR oil-based formulations. These results indicated the excellent whitening efficacy and transdermal delivery potential of this formulation. **Conclusions**: The combination of TPGS-based DES and self-assembly technology represents a revolutionary approach for advanced transdermal delivery and the development of skin care products.

## 1. Introduction

Skin whitening has become an increasingly significant demand in daily life, driven by the need to address increased melanin amount caused by hyperpigmentation, senile lentigines, and post-inflammatory pigmentation [1]. However, traditional whitening ingredients such as phenylethyl resorcinol, arbutin, kojic acid face numerous challenges in practical applications. These include limited whitening efficacy, chemical instability, strong skin irritancy potentially leading to allergies or inflammation, and poor transdermal absorption [2,3]. The latter is particularly problematic due to factors such as large molecular weight and un-suitable lipophilicity, which hinder their effective penetration through the skin barrier [4], thereby restricting their whitening effects. Among the emerging whitening agents, 4-butylresorcinol (4-BR), a 4-substituted derivative of resorcinol, has been widely recognized as a “highly effective resorcinol derivative that inhibits both tyrosinase and tyrosinase-related protein-1 (TRP-1) [5,6]”. Notably, 4-BR is by far the most potent inhibitor of human tyrosinase, with an IC50 of 21 μmol/L, demonstrating 24 times greater efficacy than kojic acid and over 300 times than arbutin [3]. D-α-Tocopheryl polyethylene glycol 1000 succinate (TPGS), an FDA-approved pharmaceutical excipient and vitamin E derivative, exhibits well-documented antioxidant properties that mitigate oxidative stress and functions as an inhibitor of P-glycoprotein (P-gp)-mediated drug efflux, thereby enhancing the efficacy of active pharmaceutical ingredients (APIs) [7,8].

Deep Eutectic Solvents (DES), a novel solvent system formed by non-covalent interactions such as hydrogen bonding between two or more components, have gained significant attention in drug delivery and cosmetic applications due to their low melting points, simple preparation, cost-effectiveness, and environmental friendliness [9,10]. DES incorporating APIs as components are particularly noteworthy, as they not only enhance API solubility and stability but also optimize intermolecular interactions, offering a better penetration for APIs. Bin Li et al. formulated an amphiphilic DES using natural oxymatrine and lauric acid, which enhanced the penetration and retention of triamcinolone acetonide, demonstrating significant anti-psoriasis therapeutic efficacy and biocompatibility [11].

DES-based carrier-free nanoparticles, composed exclusively of functional molecules without the inclusion of inert excipients, demonstrate significant potential in enhancing drug delivery efficacy. By leveraging the intrinsic properties of deep eutectic solvents (DESs) and eliminating traditional carriers, these formulations not only achieve superior drug penetration through the establishment of a high concentration gradient on the skin surface but also minimize excipient-related side effects, such as skin irritation and potential toxicity [11,12]. Furthermore, the absence of inert carriers enhances drug loading capacity and stability, making DES-based carrier-free nanoparticles a promising approach for optimizing therapeutic outcomes while reducing adverse effects.

This study aims to integrate DES technology with nanotechnology to overcome the limitations of 4-BR in skin-whitening applications. A novel “pregnant molecule”-like DES structure was fabricated composed by whitening agent 4-BR and the surfactant TPGS. Subsequently, a carrier-free 4-BR/TPGS DES nanoparticle system was prepared through self-assembly method. The synthesized DES and carrier-free 4-BR/TPGS DES nanoparticle was characterized in this study.

## 2. Results and Discussion

### 2.1. Properties of 4-BR/TPGS DES

**Figure 1:**
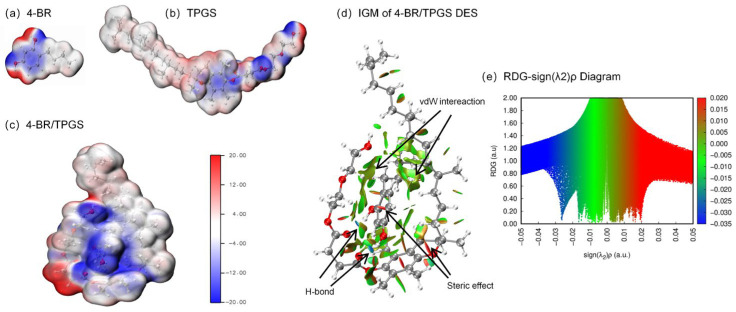
DFT calculations of 4-BR/TPGS DES with its compotent. Electrostatic potential (ESP) of (**a**) 4-BR, (**b**) TPGS, (**c**) 4-BR/TPGS DES. (**d**) The corresponding color-filled isosurface through the independent gradient model based on Hirshfeld partition (IGMH) analysis and (**e**) Scatter plots between RDG vs. Sign (λ2)ρ of 4-BR/TPGS DES.

The results of the DFT calculations for 4-BR/TPGS DES and its components are presented in Figure 1. ESP maps are depicted for 4-BR (a), TPGS (b), and 4-BR/TPGS DES (c). The ESP provides information about the charge distribution on the molecular surfaces. In the case of 4-BR and TPGS, distinct ESP patterns are observed. The color depth in the molecular surface illustrated.

The intensity of ESP. As shown in Figure 1a, the negative ESP regions of 4-BR are located on the oxygen atoms, while the positive ESP regions of TPGS are mainly located on the hydrogen atoms of the hydroxyl groups in the polyethylene glycol (PEG) chain. When 4-BR and TPGS combine to form the 4-BR/TPGS DES, the positive regions of TPGS are attracted to the negative regions of 4-BR, leading to electron transfer and delocalization [13].

Notably, in the structure of the formed 4-BR/TPGS DES, 4-BR is encapsulated by TPGS, resembling a “pregnant molecule”. This unique structural formation can be attributed to multiple factors, such as hydrogen-bonding, van der Waals forces and other intermolecular forces. The non-polar parts of 4-BR and TPGS interact through these forces, further facilitating the encapsulation process. The RDG analysis of 4-BR/TPGS DES (Figure 1d) further reveals the nature of the intermolecular interactions. The RDG isosurfaces colored by sign(λ2)ρ (Figure 1e), show different types of interactions. The green regions in the RDG isosurfaces indicate van der Waals (vdW) interactions, the specific blue patterns in the RDG analysis indicate the presence of H-bonds, as the black arrows point out the regions of hydrogen-bond, vdW interactions and steric effects. These various intermolecular interactions work together to contribute to the overall stability of the 4-BR/TPGS DES.

This “pregnant molecule”-like structure, where TPGS encapsulates 4-BR, has potential to inhibit the oxidation of 4-BR. As Figure 1c, the encapsulation by TPGS provides a physical barrier around 4-BR, reducing the exposure of 4-BR to oxygen in the environment. Oxidation of 4-BR occurs when it reacts with oxygen, the reduced oxygen accessibility has potential to slows down the oxidation process. Additionally, the transfered electronic environment may also affect the reactivity of 4-BR towards oxygen. The low-ESP-value center formed by the benzene rings may stabilize the electrons in 4-BR, making it less likely to donate electrons to oxygen and thus less prone to oxidation [14]. Further experiments will be conducted to validate this hypothesis.

**Figure 2:**
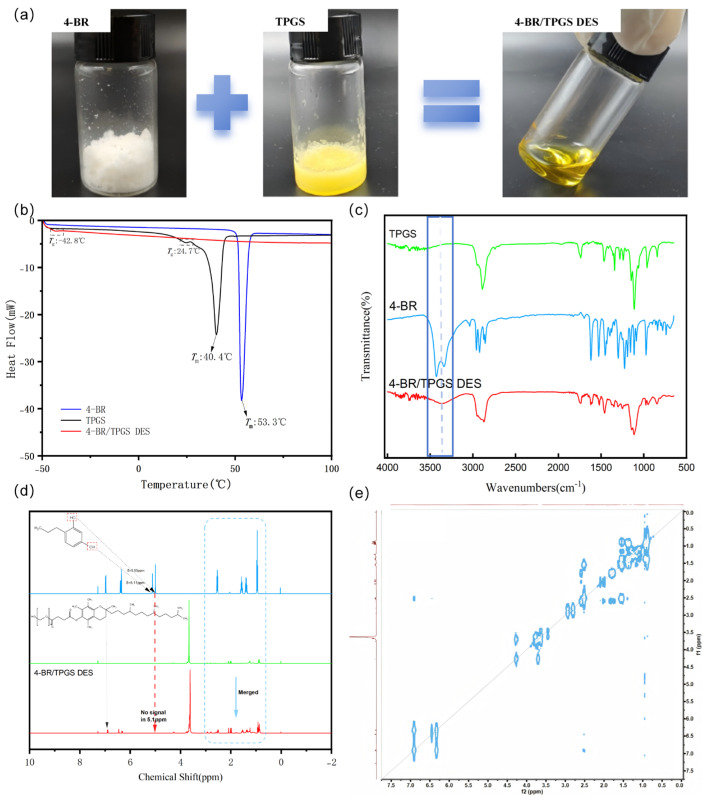
(**a**) Characterization of 4-BR, TPGS, and 4-BR/TPGS DES. (**b**) Differential scanning calorimetry (DSC) thermograms (**c**) Fourier transform infrared (FTIR) spectra (**d**) Proton nuclear magnetic resonance (^1^H NMR) spectra (400 MHz, CDCl3) and (**e**) 2D ^1^H^-1^H Correlation Spectroscopy (COSY) spectra (400 MHz, CDCl3) of 4-BR/TPGS DES and its components.

4-BR and TPGS were successfully combined to prepare 4-BR/TPGS DES at a molar ratio of 1:1, as illustrated in Figure 2a. The white powder of 4-BR and the yellowish waxy solid of TPGS transformed into a homogeneous, yellow-colored liquid DES, indicating the successful formation of 4-BR/TPGS DES. The possible formation mechanism, molecular structure, and interaction relationships of 4-BR/TPGS DES were investigated using DSC, FTIR, ^1^H-NMR, and 2D NOESY spectra techniques (Figure 2b–e).

DSC analysis, as shown in Figure 2b, provided insights into the thermal properties of 4-BR/TPGS DES and its components. Pure 4-BR exhibited a melting point at approximately 53.3 °C, while TPGS melted at 40.4 °C. However, for the 4-BR/TPGS DES, no distinct melting point was observed within the temperature range of −50 to 100 °C. The absence of a melting point confirms the successful formation of 4-BR/TPGS DES. Intermolecular interactions, such as hydrogen bonds and van der Waals forces (as depicted in Figure 1d), likely contribute to this thermal behavior. These interactions prevent the DES from undergoing a phase transition to the liquid state within the tested temperature range, which is a characteristic feature of DES.

The presence of molecular interactions in 4-BR/TPGS DES and its components were then detected by FTIR spectra, as shown in Figure 2c, hydroxyl(-OH) stretching vibration peaks of 4-BR were detected in the range of 3300–3500 cm^−1^, and the C-H stretching vibration peaks of the saturated hydrocarbon chains in TPGS within the range of 2800–3000 cm^−1^ were also detected. These peaks exhibited notable changes as the formation of 4-BR/TPGS DES. The C-H stretching vibration peaks showed a characteristic shift (e.g., 2884.36→2868.66 cm^−1^). The individual hydroxyl stretching vibration signals of 4-BR weakened and merged into a single, broad peak (highlighted in the blue box). These changes may attribute to formation of strong hydrogen bond between phenolic hydroxyl of 4-BR and the oxygen in the ether bond of TPGS. the proton between 4-BR and TPGS was “shared” and more on the side of 4-BR [15]. Similar results were reported by Bin et al. and Xing et al., respectively in OMT-LCFA DESs and between cocrystals of naproxen and oxymatrine [15,16].

In the ^1^H-NMR analysis, as shown in Figure 2d, the 4-BR spectrum initially displayed a distinct proton phenolic hydroxyl signal at a chemical shift of approximately 5.10 ppm. However, upon the formation of 4-BR/TPGS DES, this peak disappeared entirely rather than simply overlapping with other peaks (dotted squares in Figure 2d). This phenomenon suggested that a strong hydrogen bond may occur between phenolic hydroxyl of 4-BR and TPGS [17]. The phenolic hydroxyl group of 4-BR acts as a hydrogen-bond donor, interacting with specific functional groups in TPGS (such as the aldehyde group). This interaction leads to a significant change in the local proton environment of the phenolic hydroxyl group, causing the characteristic phenolic hydroxyl peak to vanish in the NMR spectrum. COSY spectra also shows there has no ^1^H-^1^H Correlation interactions at the chemical shift of 5.10 ppm.

The combined results from DSC, FTIR, and 1H-NMR analyses strongly support the successful synthesis of 4-BR/TPGS DES. Furthermore, the 4BR and TPGS could also form DES successfully at molar ratio 2:1 and 1:2 (Figure S1). The observed changes in the NMR, FTIR, and DSC characteristics provide in-depth insights into the molecular-level interactions, structure formation, and thermal properties of the DES, confirming the formation of a new DES with distinct properties compared to its individual components.

### 2.2. Properties of 4-BR/TPGS DES NPs

**Figure 3:**
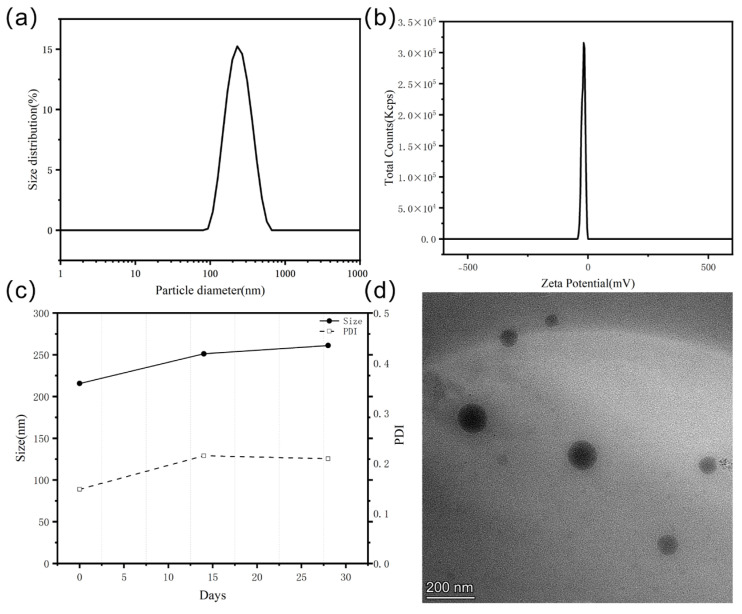
Characterization of 4–BR/TPGS DES NPs: (**a**) Particle size distribution; (**b**) Zeta potential; (**c**) Stability over 30 days (size and PDI); (**d**) TEM image (scale bar: 200 nm).

As depicted in Figure 3a, the 4-BR/TPGS DES NPs exhibited a well-defined particle size distribution with an average particle size of 216.9 nm, indicating a relatively small and potentially favorable size for applications of transdermal drug delivery [18]. The polydispersity index (PDI) was 0.1667, which suggested a relatively narrow size distribution and a more homogeneous population of nanoparticles. The zeta potential of the 4-BR/TPGS DES NPs, as shown in Figure 3b, was approximately −19.41 mV, implies the presence of electrostatic repulsion between the nanoparticles. This electrostatic repulsion is beneficial as it helps to prevent particle aggregation, thereby ensuring the long-term stability of the nanoparticle suspension (Figure 3c). The TEM image in Figure 3d offered a visual assessment of the particle morphology and size of 4-BR/TPGS DES NPs.

Notably, the average particle size observed from the TEM analysis was slightly smaller than that determined by DLS. The TEM-measured average particle size was approximately 150 nm, in contrast to the 216.9 nm obtained from DLS, representing a reduction of approximately 30%. This discrepancy is likely attributed to the water evaporation that occurs during the TEM sample-preparation process and the electron-based observation procedure. Despite this size difference, the TEM analysis still revealed that the 4-BR/TPGS DES NPs had a relatively uniform particle size distribution and a spherical or near-spherical shape, which be beneficial for transdermal delivery.

Regarding the long-term stability, Figure 3c shows the size and PDI of the 4-BR/TPGS DES NPs over a period of 30 days. Results indicated that the particle size increased slightly over time, but remained within an acceptable range (less than 300 nm). The PDI also showed a minor change, suggesting that the size distribution of the NPs remained relatively stable, which is essential for their practical applications in drug delivery and cosmetic formulations.

Overall, the combination of appropriate particle size, narrow size distribution, significant zeta potential, and good long-term stability makes the 4-BR/TPGS DES NPs a promising candidate for the application in whitening.

**Figure 4:**
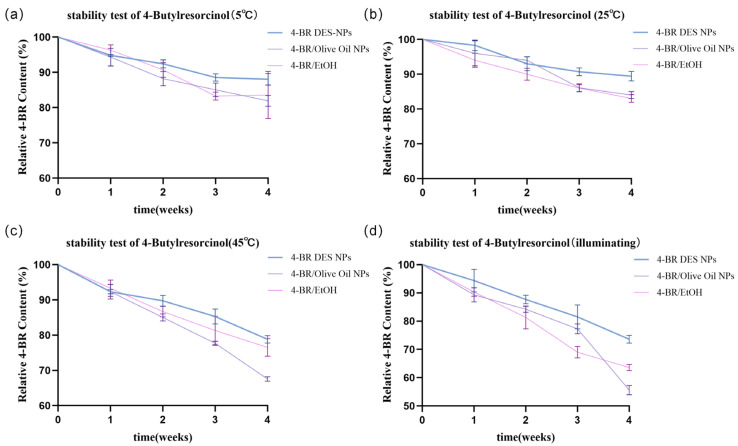
Stability assessment of 4-BR in different formulations (4-BR DES NPs, 4-BR/Olive Oil NPs, and 4-BR/EtOH) under various storage conditions: (**a**) 5 °C, (**b**) 25 °C, (**c**) 45 °C, and (**d**) illumination. The plots depict the change in relative 4-BR concentration (%) over a period of 4 weeks, with error bars representing the standard.

As depicted in Figure 4, the stability of 4-BR in three formulations (4-BR DES NPs, 4-BR/Olive Oil NPs, and 4-BR/EtOH) was systematically evaluated under various storage conditions to assess its long-term stability for pharmaceutical and cosmetic applications. At 5 °C and 25 °C (Figure 4a,b), 4-BR DES NPs showed no significant differences from the other two groups, with concentration differences within 5%. However, under 45 °C and illumination, distinct disparities emerged. After four weeks at 45 °C (Figure 4c), the 4-BR concentration in 4-BR/Olive Oil NPs and 4-BR/EtOH decreased notably, while that in 4-BR DES NPs declined less. Specifically, the decreased 4-BR concentration in 4-BR/Olive Oil NPs was 1.53-fold higher than that in 4-BR DES NPs and 1.38-fold higher than that in 4-BR/EtOH. This indicates 4-BR DES NPs better preserve 4-BR integrity under high-temperature conditions. Similarly, under illumination (Figure 4d), 4-BR DES NPs demonstrated superior stability. After four weeks of light exposure, the 4-BR concentration in 4-BR DES NPs was 1.32-fold higher than in 4-BR/Olive Oil NPs and 1.15-fold higher than in 4-BR/EtOH, highlighting the enhanced stability of 4-BR in 4-BR DES NPs.

The improved stability of 4-BR in DES NPs may be attributed to several mechanistic factors, as illustrated in Figure 1c. The “pregnant molecule”-like structure of the DES plays a pivotal role in this context. TPGS, a key component of this structure, exhibits potent antioxidant properties, effectively scavenging free radicals that could otherwise induce the oxidation of 4-BR [19]. Furthermore, the unique architecture of the 4-BR/TPGS DES provides dual protection—both physical and electronic—for 4-BR. Physically, the DES structure may shield 4-BR from detrimental environmental factors such as oxygen and light. Electronically, it modulates the electronic environment surrounding 4-BR, thereby inhibiting its oxidation.

### 2.3. In Vitro Permeation Experiment

**Figure 5:**
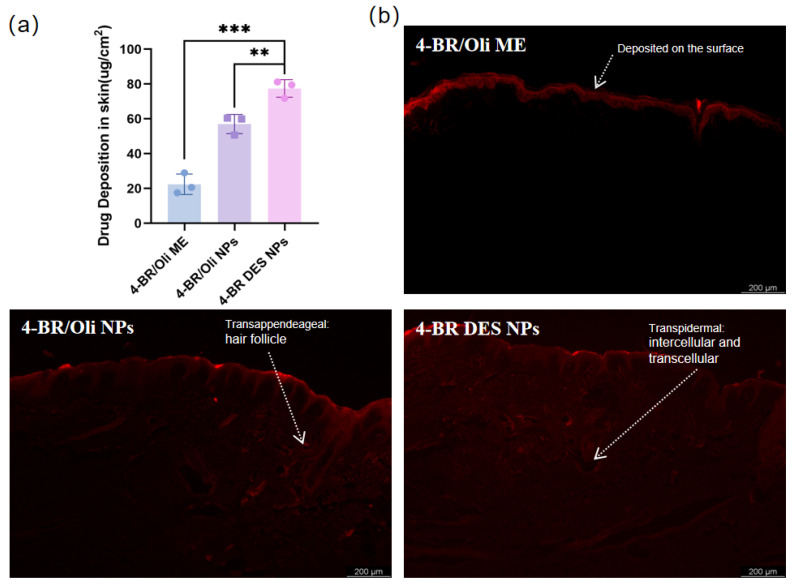
(**a**) Drug deposition in Bama miniature pigs after 4 h exposure to three formulations: 4-BR/Olive Oil ME, 4-BR/Olive Oil NPs, and 4-BR DES NPs. Data expressed as mean ± SD (n = 3). (**b**) Representative fluorescence micrographs showing dermal penetration profiles of butyl rhodamine B-loaded formulations; white scale bars = 200 μm. Statistical significance is indicated by asterisks (** *p* < 0.01, *** *p* < 0.001).

Given the significant barrier function of the stratum corneum (SC) that impedes drug transport [18], Franz diffusion cells and in-vitro models offer a direct and effective approach for evaluating the efficacy of transdermal drug delivery. In this part, fluorescence penetration experiments were carried out to assess the transdermal absorption of 4-BR in different formulations, namely 4-BR/Olive Oil ME, 4-BR/Olive Oil NPs, and 4-BR/TPGS DES NPs. As shown in Figure 5b, the fluorescence results revealed distinct differences among the formulations. The fluorescence of 4-BR/Olive Oil ME was mainly accumulated on the surface of the skin, indicating limited penetration. In contrast, the 4-BR DES NPs demonstrated the ability to penetrate through the stratum corneum and reach deeper layers of the skin, suggesting superior transdermal performance.

The drug deposition data in Figure 5a further supported these findings. The amount of 4-BR deposited per unit area in the 4-BR DES NPs group was 77.49 μg/cm^2^, which was 3.46-fold higher than that in the 4-BR/Olive Oil ME group and 1.36-fold higher than that in the 4-BR/Olive Oil NPs group, representing a 35.8% increase compared to the 4-BR/Olive Oil NPs group. Similar conclusions were drawn by Bin et al. and Xu et al., for they respectively achieved a 66.83 ± 2.89 μg/cm^2^ TA transdermal and 4.3 times Retinol deposition concentration in skin [11,20]. The enhanced transdermal absorption of 4-BR in the 4-BR DES NPs formulation might be attributed to (1) the solubilizing effect through micelle formation mediated by TPGS. Borgheti et al. reached a 2.1-fold concentration of transdermal nicotine delivery for the use of TPGS as a surfactant [21]. While for the 4-BR/Oli NPs, with a well-known Tween 80 as surfactant, 4-BR DES NPs still have 1.36-fold higher than it, implies other potential mechanisms such as (2) Lipid extraction and lipid fluidization; Interaction with intracellular keratin; increasing partitioning into the skin and et, Which were mainly related to the “brick and molar” structure of SC [10].

In summary, these findings suggest that DES combined with nanoparticle technology could effectively penetrate the SC and reach the deeper layers of the skin. Accordingly, 4-BR/TPGS DES NPs could be more effective and potentially facilitate the targeted delivery of 4-BR to the dermis.

### 2.4. Whitening Activity of 4-BR/TPGS DES NPs 

**Figure 6:**
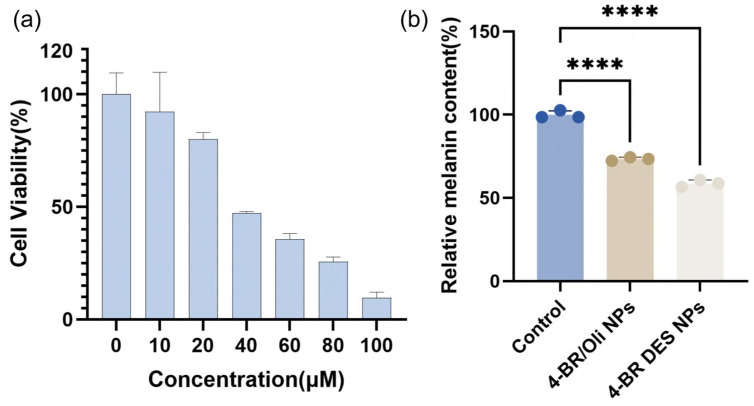
(**a**) Cell viability assay of B16 cells treated with 4-BR DES NPs containing different concentrations of 4-BR (0–100 μM). (**b**) Relative melanin content in B16 cells after 24-h treatment with various methods: control (blank), 4-BR/Oil NPs (15 μM 4-BR) and 4-BR DES NPs (15 μM 4-BR). Statistical significance is indicated by asterisks (**** *p* < 0.0001).

The whitening activity of 4-BR in different formulations was evaluated using B16 cells and zebrafish models. In Figure 6a, the CCK-8 assay results for B16 cells treated with 4-BR DES NPs containing different concentrations of 4-BR (0–100 μM) showed a concentration-dependent decrease in cell viability, with an IC50 value of 47.05 μM 4-BR. At lower concentrations (0–20 μM), cell viability remained relatively high, above 80%, indicating minimal cytotoxicity. However, as the concentration increased to 100 μM, cell viability dropped to 9.67%, suggesting potential toxicity at high concentrations. Based on these results, a 15μM concentration of 4-BR with 85% cell availability, was selected for subsequent experiments.

The relative melanin content in B16 cells after 24-h treatment with various formulations is presented in Figure 6b. Compared to the control group, both 4-BR/Oil NPs and 4-BR DES NPs significantly reduced the melanin content. Specifically, the melanin extraction amount in B16 cells incubated with 4-BR/Oil NPs was 73.37% of that in the control group, while for the 4-BR DES NPs group, it was 58.7% of the control. This indicates that 4-BR DES NPs led to a more substantial reduction in melanin content in B16 cells. In terms of inhibitory effect, 4-BR DES NPs exhibited a 1.55-fold higher inhibition compared to 4-BR/Oil NPs at the same 4-BR concentration (15 μM).

**Figure 7:**
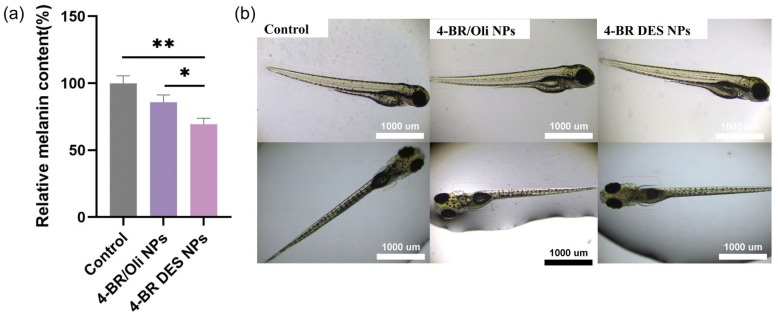
(**a**) Relative melanin content in zebrafish following treatment with different methods. (**b**) Representative images of zebrafish after 72-h incubation. Statistical significance is indicated by asterisks (* *p* < 0.05, ** *p* < 0.01).

In the zebrafish model, Figure 7a shows the relative melanin content after treatment with different formulations. Similar to the results in B16 cells, both 4-BR/Oil NPs and 4-BR DES NPs reduced the melanin content in zebrafish. The 4-BR/Oil NPs group showed a significant reduction of 14.1% compared to the control (*p* < 0.05), while for the 4-BR DES NPs group had a 30.6% reduction (*p* < 0.01), which was 2.16-fold higher than that of 4-BR/Oil NPs group. The representative images of zebrafish in Figure 7b further visually confirmed these findings. The zebrafish treated with 4-BR DES NPs appeared lighter in color compared to the control and 4-BR/Oil NPs treated groups, especially in the back of zebrafish larvae where the melanin mainly enriched [22], indicating a decrease in melanin production.

Overall, these results suggest that the 4-BR DES NPs formulation exhibits enhanced whitening activity both in vitro (B16 cells) and in vivo (zebrafish) compared to 4-BR/Oil NPs at the same concentration of 4-BR. The following reasons may explain this phenomenon:

(1) Properties of TPGS (a component of 4-BR/TPGS DES): As an FDA-approved pharmaceutical excipient, TPGS is a derivative of vitamin E with well-documented antioxidant properties and the ability to mitigate oxidative stress. Additionally, TPGS acts as an inhibitor of P-glycoprotein (P-gp)-mediated drug efflux. For instance, Pankaj Kumar Sharma et al. reviewed the antioxidant properties of TPGS in oxidative stress-mediated ocular diseases and its role as a P-gp inhibitor, which can prevent nasolacrimal drainage of drugs [6]. Similarly, Muhammad Asim Farooq et al. summarized that TPGS may bind to the non-transport active sites of P-gp, leading to conformational changes and disrupting its transport function, thereby enhancing its anticancer effects [23]. Given that elevated oxidative stress, higher ROS levels, increased intracellular accumulation of 4-BR (due to P-gp-mediated efflux inhibition) in B16 cells can lead to enhanced melanin production, the antioxidant and P-gp inhibitory properties of TPGS may contribute to the observed whitening effects [24,25]. Although there are currently no direct reports on the effects of TPGS on B16 cells and zebrafish, the aforementioned mechanisms provide a plausible explanation for the observed phenomenon.
(2) Higher cellular uptake rate: The higher cellular uptake rate of 4-BR DES NPs may also contribute to their enhanced whitening activity [26]. The primary distinction between 4-BR/TPGS DES NPs and 4-BR/Oil NPs lies in their formulation composition. For the former, the melanin-inhibiting component 4-BR is dissolved with TPGS in a DES, which enhances the solubility of 4-BR. These NPs are assembled through high-pressure homogenization, with TPGS acting as a surfactant. For the letter, 4-BR is dissolved in olive oil (due to its poor water solubility) and stabilized by the nonionic surfactant Tween 80. Similar to traditional 4-BR preparations, results in the simultaneous uptake of olive oil during cellular absorption by B16 cells and zebrafish, thereby hindering the efficient absorption of 4-BR.

## 3. Materials and Methods

### 3.1. Materials

4-Butylresorcinol (4-BR, 99%) was purchased from Zhuhai Beri Pharmaceutical Technology Co., Ltd (Zhuhai, China). TPGS (D-α-tocopherol polyethylene glycol 1000 succinate, 99%) was obtained from Guangzhou Congen Pharmatec Co., Ltd (Guangzhou, China). Deuterated chloroform (CD Cl_3_), potassium bromide (KBr), olive oil, soybean phospholipids, phosphoric acid, acetonitrile (HPLC grade) were supplied by Aladdin Reagent Co., Ltd (Shanghai, China). Carbomer was purchased from Lubrizol Corporation (Cleveland City, OH, USA). Sodium dodecyl sulfate (SDS) was obtained from Solarbio Life Sciences (Beijing, China). Paraformaldehyde (4%), phosphate-buffered saline (PBS), DMEM medium, fetal bovine serum (FBS), equivalent triple antibiotic, and trypsin were procured from Servicebio Biotechnology Co., Ltd (Wuhan, China). The CCK-8 assay kit was obtained from Meilun Biotechnology Co., Ltd (Dalian, China). All chemicals and reagents were of analytical or HPLC grade and used without further purification unless otherwise stated.

### 3.2. Methods

#### 3.2.1. Preparation of 4-BR/TPGS DES

To synthesize and characterize the 4-BR/TPGS DES, stoichiometric mixtures of 4-Butylresorcinol and TPGS were prepared at molar ratios of 1:1 (Figure 2a). The mixtures were homogenized under controlled thermal conditions (60 ± 2 °C) using a magnetic stirring hotplate at 300 rpm for 30 min to ensure complete eutectic formation. The resultant transparent liquids were cooled to 25 °C and stored in anhydrous conditions prior to further characterization.

#### 3.2.2. Theoretical Calculation Methods

The geometries of these two molecules and their complex were all optimized with dispersion corrected density functional theory (DFT-D3) at the B3LYP-D3/6-31G(d) ^12^level. All these DFT calculations were performed using Gaussian 16 program suite (Gaussian, Inc., Wallingford CT, USA, 2016) [27]. In order to analyze the intermolecular interactions, the noncovalent interaction (NCI) analysis, which was also named Reduced density gradient (RDG) analysis was performed using Multiwfn program [28] to study the weak interaction visually. The electrostatic surface potentials (ESP) were calculated using Multiwfn program version 3.8 and the mapping of ESPs were all rendered using Visual Molecular Dynamic program (VMD) version 1.9.3 [29].

#### 3.2.3. Characterization of 4-BR/TPGS DES

##### Nuclear Magnetic Resonance (NMR) Spectroscopy

NMR spectra were acquired on an AVANCE NEO 400 MHz spectrometer (Bruker, Switzerland AG, Fällanden, Switzerland) using deuterated chloroform (CDCl_3_) as the solvent. Samples were dissolved in CDCl_3_ at a concentration of 10 mg/mL, and chemical shifts were referenced to tetramethylsilane (TMS) at 0 ppm. The experiments were performed to confirm the chemical structure and interactions between 4-BR and TPGS in the DES. ^1^H-NMR spectra were recorded with a spectral width of 12 ppm, 32 scans, and a relaxation delay of 1 s. The chemical shifts were assigned based on the characteristic peaks of 4-BR and TPGS, and the characteristic changes in chemical shifts were used to reflect the intermolecular interactions in the 4-BR/TPGS DES. Specifically, the downfield or upfield shifts of proton signals were analyzed to identify hydrogen bonding, electron delocalization, or other non-covalent interactions between 4-BR and TPGS.

##### Fourier-Transform Infrared (FTIR) Spectroscopy

FTIR spectra were recorded on a Nicolet iS50 spectrometer (Thermo Fisher Scientific, Waltham, MA, USA) equipped with both transmission and attenuated total reflectance (ATR) modules. For 4-BR and TPGS, the sample was homogenized with spectroscopic-grade potassium bromide (KBr, Merck, Darmstadt, Germany) at a 1:100 (*w*/*w*) ratio and pressed into a translucent pellet under 10-ton hydraulic pressure. Background subtraction was performed using a blank KBr pellet. For 4-BR/TPGS DES, the diamond ATR accessory (Smart iTR™) was employed without sample pretreatment. All spectra were acquired by averaging 16 consecutive scans over the wavenumber range of 4000–400 cm^−1^ at a resolution of 4 cm^−1^. The spectra were baseline-corrected, and the characteristic peaks were assigned to specific functional groups to analyze the interactions between 4-BR and TPGS in the DES.

##### Differential Scanning Calorimetry (DSC)

DSC was performed using a METTLER TOLEDO DSC3 instrument (Zurich, Switzerland) to investigate the thermal behavior and phase transitions of 4-BR, TPGS, and 4-BR/TPGS DES. Approximately 6 mg of each sample was sealed in a standard 40 μL aluminum crucible with a pierced lid. The temperature program included a heating step from −50 °C to 100 °C under a nitrogen purge (50 mL/min), with a controlled heating rate of 10 °C/min. An empty aluminum pan served as the reference The glass transition temperature (Tg), melting temperature (Tm), and enthalpy changes (∆H) were determined from the DSC thermograms. The data were analyzed using STARe software version 14 to evaluate the thermal stability and compatibility of the DES components.

#### 3.2.4. Preparation of 4-BR/TPGS DES Nanoparticles (4-BR/TPGS DES NPs)

4-BR/TPGS DES NPs were prepared using self-assembly method. Briefly, 4-BR/TPGS DES (5.0 wt%) and aqueous phase was formulated with deionized water (95 wt%). The deep eutectic solvent phase was gradually incorporated into the aqueous phase under controlled shear mixing (625 rpm, 15 min) using an IKA RW20 Digital overhead stirrer (Staufen, Germany) and formed 4-BR/TPGS DES NPs. The final 4-BR/TPGS DES NPs dispersion appeared as a pale-yellow milky emulsion, which was stored at 4 °C for subsequent characterization.

#### 3.2.5. Preparation of Olive-Oil Based Microemulsion (4-BR/Olive Oil ME)

The olive oil based 4-Butylresorcinol microemulsion (4-BR/Olive Oil ME) was formulated as a comparative formulation. While maintaining identical 4-BR concentration (10 mg/mL) to the 4-BR/TPGS DES NPs, the surfactant system was modified by replacing TPGS with soybean phospholipids (2.0% *w*/*w*, 2000 mg) and oleic acid (0.8% *w*/*w*, 800 mg) to optimize interfacial stability, and the oil phase was replaced by Olive Oil (10 wt%) to dissolve 4-BR. To prevent drug precipitation, 0.15 wt% Carbomer 940 was incorporated as a viscosity-enhancing agent. The final 4-BR/Olive Oil ME appeared as a pale milky cream.

#### 3.2.6. Preparation of Olive-Oil Based Nanoparticles (4-BR/Olive Oil NPs)

The materials and preparation process for 4-BR/Olive Oil NPs were identical to those for 4-BR/TPGS DES NPs, in which the 4-BR/TPGS DES were replaced by the same concentration of 4-Butylresorcinol dissolved in Olive Oil (10 wt%), resulting in the production of a pale, opaque emulsion.

#### 3.2.7. Stability Test of 4-BR in Above Formulations

The stability of 4-BR in three formulations (4-BR DES NPs, 4-BR/Oli NPs, and 4-BR/EtOH) (1 g of 4-BR dissolved in 100 mL EtOH) was evaluated under accelerated storage conditions to assess preliminary stability characteristics. The samples were divided into four sets and stored under the following conditions for 4 weeks. (1) 5 °C; (2) 25 °C; (3) 45 °C; and (4) Illumination. This short-term study focused on temperature and light effects as primary degradation factors based on ICH Q1A(R2) guidelines for accelerated stability testing. At weekly intervals, 4-BR concentration was quantified by high–performance liquid chromatography (HPLC) using a Vanquish HPLC system (Thermo Fisher Scientific) equipped with a Waters Symmetry C18 column (4.6 × 250 mm, 5 μm). The mobile phase consisted of acetonitrile and 0.1% phosphoric acid (65:35, *v*/*v*) delivered at a flow rate of 1.0 mL/min. Detection was performed at a wavelength of 210 nm. Samples were diluted 100–fold prior to injection, and the injection volume was 20.0 µL. The concentration of 4–BR was determined against a calibration curve, with triplicate measurements performed for each sample. The retention time for 4–BR under these conditions was 3.77 min.

### 3.3. Characterizations of 4-BR/TPGS DES NPs

#### 3.3.1. Size, Zeta Potential and TEM of 4-BR/TPGS DES NPs

The average particle size (Z-average), polydispersity index (PDI), and zeta potential of the nanoparticles were determined by dynamic light scattering (DLS) and electrophoretic light scattering (ELS) using a Malvern Zetasizer Nano ZS90 (Malvern Panalytical, Malvern, UK) equipped with a DTS1070 zeta potential cell. Prior to measurement, the NPs dispersion was diluted 200-fold with ultrapure water (18.2 MΩ·cm) to achieve optimal scattering intensity. Triplicate measurements were performed at 25 °C with a 2-min equilibrium period for each sample. Nanoparticle morphology was examined using a Talos F200X G2 TEM (Thermo Fisher Scientific, USA) operated at an accelerating voltage of 200 kV. For imaging, a 5 μL aliquot of NPs was deposited onto a carbon-coated copper grid (200 mesh, Zhongjingkeyi Technology Co., Ltd., Beijing, China). After air-drying for 10 min, the grid was loaded into the TEM for imaging.

#### 3.3.2. Ex Vivo Permeation Studies

The transdermal permeation study was conducted using a TP-6 transdermal diffusion system (Light Equipment, Tianjin, China) equipped with vertical Franz-type diffusion cells (orifice diameter 15 mm, Light Equipment, Tianjian, China). Each cell consisted of a 15 mL receptor compartment filled with physiological saline containing 0.1% (*v*/*v*) Tween 80 (pH 7.4 ± 0.2) to maintain the leakage condition and simulate a realistic skin permeation process, and a 1.77 cm^2^ effective diffusion area (1.5 cm diameter circular exposure window). Excised skin specimens from Bama miniature pigs (Guangquan Biotechnology, Jinan, China) with intact stratum corneum were mounted between the donor and receptor chambers with the stratum corneum facing upward, ensuring bubble-free interfacial contact. The skin specimens were obtained from adult Bama miniature pigs, and the age of the animals was not specified. The skin preparation process involved careful removal of subcutaneous fat and connective tissue, followed by rinsing with physiological saline to ensure cleanliness and integrity. The prepared skin was stored at −20 °C until use and thawed at room temperature before each experiment. 4-BR/TPGS DES NPs, 4-BR/Olive Oil NPs and 4-BR/Olive Oil ME were applied uniformly (2 mL) to the donor compartment. The receptor phase was continuously agitated at 350 rpm using a magnetic stir bar, utilizing the integrated thermostatic circulation system of the TP-6 transdermal apparatus to simulate physiological temperature conditions at 32 ± 0.5 °C. All experiments were performed in triplicate under light-protected conditions for 4 h for full execution.

#### 3.3.3. In Vitro Assessment of Quantitative Permeation Using Intradermal Retention Test

The quantitative assessment of in vitro intradermal retention was performed using an optimized extraction protocol. In brief, Excised skin specimens from Bama miniature pigs were sectioned into 1.5 × 1.5 mm^2^ pieces and immersed in 10 mL of HPLC-grade acetonitrile. Samples were first subjected to ultrasonication at 40 kHz (TAISITE, Tianjin, China) for 10 min to mechanically disrupt the stratum corneum microstructure through cavitation effects [30], followed by mechanical agitation at 200 rpm for 12 h at 25 °C to ensure complete compound release. The extracts were filtered through 0.22 μm hydrophobic PVDF syringe filters (Biosharp, Beijing, China) and analyzed via a Vanquish HPLC system (Thermo Fisher Scientific, Waltham, USA) (method 2.2.7).

#### 3.3.4. In Vitro Assessment of Qualitative Permeation Using Inverted Fluorescence Microscope

The qualitative assessment of in vitro intradermal retention was conducted using Franz diffusion cells. Samples, spiked with an equivalent concentration of butyl rhodamine B as a fluorescent marker, were applied to donor chamber and diffuse for 1 h to visualize and quantify dermal deposition. The skin samples were incubated for a predetermined period, after which they were collected and preserved in 4% paraformaldehyde overnight. Subsequently, the samples were cleansed with phosphate-buffered saline and promptly frozen at −80 °C. The frozen samples were vertically immersed in the OCT compound. Whole-layer slices were acquired using a freeze slicer (Leica CM3050S, Leica, Wetzlar, Germany) and visualized using a cryostat, and fluorescence imaging was performed using a Leica DMI-8 manual inverted fluorescence microscope equipped with appropriate filter sets for butyl rhodamine B detection. Representative images were captured for qualitative analysis of intradermal distribution patterns.

### 3.4. Whitening Activity Assessment

#### 3.4.1. Cell Culture

To assess the whitening activity of the formulations and evaluate the potential of 4-BR/TPGS DES NPs for melanin inhibition, the spindle-shaped and epithelial-like murine melanoma cell line B16-F10 Cells-a widely adopted model in cosmetic research due to their high melanogenic capacity and conserved melanogenesis pathways-were maintained in a standard incubator at 37 °C with 5% CO_2_ atmosphere. The culture medium consisted of high-glucose DMEM supplemented with 10% fetal bovine serum (FBS) and 1% antibiotic-antimycotic solution (penicillin-streptomycin-amphotericin B).

#### 3.4.2. Cell Viability Assay of 4-BR/TPGS DES NPs in B16 Cells

To evaluate the cytotoxicity of the formulation, B16-F10 cells were seeded in a 96-well plate at a density of 5 × 10^6^ cells/mL (100 µL per well). After cell attachment and reaching 60% confluency, the medium was aspirated, and each well was washed with 100 µL of PBS. Subsequently, 100 µL of drug-containing medium was added to each well. Upon reaching 90% confluency, the medium was replaced with fresh complete medium, and 10 µL of CCK-8 solution was added to each well. After 1 h of incubation at 37 °C, the absorbance was measured at 450 nm. The critical concentration showing no significant cytotoxicity was selected for further melanin inhibition experiments.

#### 3.4.3. Detection of Melanin Content in B16 Cells

B16-F10 murine melanoma cells were seeded at a density of 1–1.6 × 10^6^ cells per well in 6-well plates and incubated for 24 h to allow attachment. After attachment, cells were co-treated with α-MSH (1 µM) and test formulations of 4-BR (15 µM) dissolved in fresh medium. Cells were then incubated for an additional 24 h. Following incubation, cells were washed twice with ice-cold phosphate-buffered saline (PBS). Subsequently, 500 µL of trypsin (0.25%) was added to each well for cell detachment (1 mL sufficient for 3 wells). The resulting cell suspension was centrifuged at 2000 rpm for 5 min at 4 °C (Centrifuge 5425 R, Eppendorf, Hamburg, Germany), and the supernatant discarded. Cell pellets were lysed in 500 µL of alkaline lysis solution (1 mol/L NaOH, 10% DMSO) and incubated at 80 °C for 60 min in a metal bath (DLAB HB120-S, Beijing, China).

Lysates were centrifuged at 4000 rpm for 10 min to pellet debris. The supernatant was transferred to fresh tubes. Blank controls contained lysis solution without cells. After cooling to room temperature, supernatants were aliquoted in quadruplicate into 96-well plates. Absorbance was measured at 450 nm using a microplate reader (BioTek Epoch 2, Agilent, Santa Clara, CA, USA). Relative melanin content was calculated using the following formula:

(1) $$Relative\;Melanin\;Content=\frac{A_{450(sample)}-A_{450(blank)}}{A_{450(control)}}$$

where:

A_450(sample):_ Absorbance of experimental group at 450 nm

A_450(blank):_ Absorbance of blank (lysis solution without cells)

A_450(control):_ Absorbance of untreated control group (α-MSH only, no formulations)

#### 3.4.4. Detection of Melanin Content in Zebrafish

To further evaluate melanin inhibition efficacy, wild-type zebrafish (AB strain) embryos at 8 h post-fertilization (8 hpf) were incubated in 6-well plates with 5 mL per well of test formulations containing 15 μM 4-BR (equimolar concentration across experiment groups, diluted with Holt-Buffer solution) for 72 h under standard culture conditions (28.5 ± 0.5 °C), with triplicate wells per treatment group [31,32]. Post-treatment, embryos were rinsed twice with Holt-Buffer solution, transferred to 1.5 mL microcentrifuge tubes (≈15 embryos/tube), and homogenized with 150.0 μL sodium deoxycholate (5.0 mg/mL) via vortex mixing at 4 °C followed by ultrasonic disruption (200 W, 5 s pulse/5 s interval, 6 cycles) on ice (SCIENTZ-IID, Ningbo, China). Lysates were centrifuged at 10,000× *g* for 5 min (4 °C), after which the supernatant was carefully aspirated; the resultant pellet was subsequently dissolved in 150.0 μL NaOH (1.0 mol/L) with 10% DMSO using water bath sonication (40 kHz, 10 min) until complete dissolution. Supernatants were then transferred into 96-well plates. Absorbance was measured at 405 nm using a microplate reader. Relative melanin content was calculated using the following formula:

(2) $$Relative\;Melanin\;Content=\frac{A_{405(sample)}-A_{405(blank)}}{A_{405(control)}}$$

where:

A_405(sample):_ Absorbance of experimental group at 450 nm

A_405(blank):_ Absorbance of blank (1.0 mol/L NaOH with 10% DMSO)

A_405(control):_ Absorbance of untreated control group

### 3.5. Statistical Analysis

Data are presented as mean ± standard deviation (SD). Statistical significance of differences was determined by one-way ANOVA, with significance levels defined as: * *p* < 0.05, ** *p* < 0.01, *** *p* < 0.001, **** *p* < 0.0001.

## 4. Conclusions

In this study, a “pregnant molecule”-like DES structure composed by 4-BR and TPGS was first prepared. Subsequently, a novel carrier-free 4-BR/TPGS DES NPs was formed by self-assembly method. The synthesized DES demonstrated significantly potential in protecting 4-BR from oxidation. In vitro qualitative and quantitative permeation studies revealed 3.46-fold enhanced skin permeability. Furthermore, both in vitro and in vivo assessments revealed superior whitening efficacy of 4-BR/TPGS DES NPs compared to 4-BR oil NPs. In conclusion, Combining TPGS based deep eutectic solvent technology and carrier-free nanoparticles self-assembly technology is a promising strategy for transdermal delivery and skin care product.

## Data Availability

The raw data supporting the conclusions of this article will be made available by the authors on request.

## Supplementary Materials

The following supporting information can be downloaded at: https://www.mdpi.com/article/10.3390/ph18091383/s1.
