# Supplementary material for: Self-Assembly 4-Butylresorcinol Deep Eutectic Solvent Nanoparticles for Efficient Transdermal Delivery and Whitening
Source: Pharmaceuticals (Basel). 2025 Sep 16;18(9):1383. doi: 10.3390/ph18091383 (PMC12473103; doi:10.3390/ph18091383)
Supplement: Supplementary file 1 [file pharmaceuticals-18-01383-s001.zip › pharmaceuticals-3822473-supplementary.pdf]

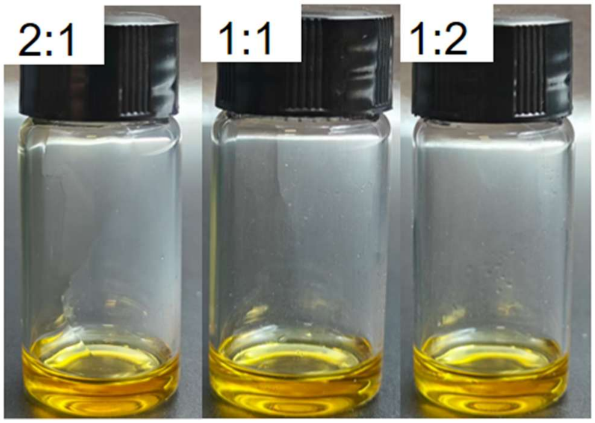

Figure S1. Digital photographs of deep eutectic solvents (DESs) composed of TPGS and 4-BR at molar ratios of 2:1, 1:1, and 1:2.

The digital images in Figure S1 depict the macroscopic appearance of DESs formulated with TPGS and 4-BR at three distinct molar ratios (2:1, 1:1, and 1:2). All samples exhibit a transparent, homogeneous liquid state—a key visual hallmark of successful DES formation. This homogeneity originates from strong intermolecular interactions (fig.1d) between TPGS and 4-BR, which synergistically depress the melting points of the individual components(fig.2b). As a result, the mixtures remain as stable liquid phases under ambient conditions, even though the molar ratio of TPGS to 4-BR varies.

The consistent transparency and lack of phase separation across all three molar ratios further imply that TPGS and 4-BR can form DESs over a range of mixing proportions. This observation highlights the robustness of the 4-BR/TPGS DES system—i.e., the two components can assemble into a uniform eutectic phase regardless of whether TPGS or 4-BR is present in excess. Such versatility in molar ratio is practically valuable for tailoring DES properties (e.g., viscosity, polarity) while maintaining the core advantage of DESs (low melting point and structural homogeneity).
